# Abstracts and Gleanings

**Published:** 1877-03-20

**Authors:** 


### Abstracts and Gleanings.

*Stimulants in Typhoid Fever.*—There are a few simple rules which may guide you in the administration of stimulants in this fever:

First.—They should never be administered indiscriminately—that is, never give a patient stimulants simply because he has typhoid fever.

Second.—When there is reasonable doubt as to the propriety of giving or withholding stimulants, it is safer to withhold them, at least until the signs which indicate their use become more marked.

Third.—In every case, but especially when stimulants are not clearly indicated, watch carefully the effect of the first few doses. There are few whose experience in the treatment of typhoid fever is such as to enable them to positively determine, from the appearance of the patient, when the administration of stimulants should be commenced.

Should you commence the administration of stimulants, it is necessary to see your patient every two hours, and note carefully the effect produced. If you find the tongue becoming dry, the patient more restless, the delirium more active, the temperature ranging higher, and the pulse more and more rapid, you may be certain that stimulants are contraindicated. If, on the other hand, the pulse becomes fuller and more regular, if the first sound of the heart is more distinctly heard, or, if it has been absent, it has returned, if the restlessness and delirium is less marked, the tongue more moist, and the patient more intelligent, you may be certain that the time for the administration of stimulants has arrived. When you have commenced their use, it is of the greatest importance that you administer them at stated intervals, especially during the night.

In a severe case of typhoid fever, a free administration of stimulants, just at a critical period (which may not last more than twenty-four hours), will often be followed by a refreshing sleep, and your patient may rapidly pass from an apparently hopeless condition to one of convalescence.

The *third* important thing to be accomplished in the management of typhoid fever patients is the maintenance of nutrition. You must bear in mind that the primary and principal effects of the typhoid poison are manifested in the changes which take place in the lymphatics of the gastro-intestinal tract. Experience has taught us that the enfeeblement of the digestive and assimilative powers, due to these glandular changes, which are manifest from the very commencement of the fever, renders the digestion of solid food impossible, and for a long time it has been the rule of the profession to allow typhoid fever patients only liquid food.

There has been and still is great diversity of opinion in regard to the special articles of diet best suited to this class of patients. Most medical writers and practitioners claim that beef-tea is the proper diet for fever patients; consequently it is the rule to pour into these enfeebled stomachs a decoction of beef in such quantities as a helthy stomach could hardly tolerate, and which in itself has little or no nutritive element.

There is no disease in which a waste of all the tissues of the body goes on so rapidly as in typhoid fever; and milk is an article of diet which furnishes the elements of nutrition necessary to repair this rapid waste, and there are not the objections to its use which there are against animal broths and gruels. Although there have been, and still are, in some quarters, strong objections against its use as an article of diet in fevers, recently it has been regarded with more favor, and those who have had most extended opportunities for testing its nutritive qualities have come to regard it as the only article of diet required by fever patients. In it we not only find all the elements required for repairing the rapidly wasting tissues, but they are in a condition to be most readily assimilated by the enfeebled digestive apparatus.

In order to make the milk more digestible, it may be diluted with lime-water. The lime-water is an antiseptic, and allays irritability of the stomach and intestines. The quantity of milk is not limited; the patient may take all his stomach will digest—usually patients will take from four to six quarts in the twenty-four hours.

After the patient has passed into the fourth week of the disease, you may find it necessary to administer cream and the yolk of eggs in connection with the milk. —*Medical Record.*

*Dilatation of the Uterus.*—Dr. Atthill, before the British Medical Association, says;

I have, therefore, in order to guard as far as possible against the serious results recorded by others as following attempts to dilate the uterus, laid down for myself the following rules, which I can recommend with confidence to others:

1. Never to dilate the cervix uteri for the cure of dysmenorrhea or sterility depending on a narrow cervical canal or conical cervix.

2. Never to dilate in cases in which a large and dense intramural fibroid presses on and partially obliterates the cervical canal.

3. Never to use metallic dilators of any kind, but to choose for the purpose either sponge or sea-tangle tents, which expand slowly and gradually.

4. Never to continue the process of dilatation for more than forty-eight hours. I prefer, in the few cases I have met with in which, after the laspe of that time, the cervix was not sufficiently opened to suit the purpose I had in view, to postpone all operative interference for some weeks rather than risk the result by prolonging the dilating process.

With respect to the first of these rules, I look upon the treatment of what is termed "mechanical dysmenorrhea" by dilatation as altogether a mistake. I doubt if any permanent benefit has ever resulted from it; while in several cases grave symptoms, and in one death, have, to my knowledge, followed the attempt. Equally, it is of importance not to prolong the dilating process. My own experience in the treatment of uterine disease requiring dilatation leads me to this conclusion, that unpleasant symptoms are likely to occur in a direct ratio to the length of time over which the process of dilatation extends. Again, I have known death to follow the attempt to dilate the uterus in a case where a large fibroid, of dense structure, giving rise to menorrhagia, and causing intense pain, was developed in the uterus, and encroached on the cervical canal. In such cases dilatation is doubly objectionable, because the process is useless as well as dangerous; useless, because you will generally find that any attempt at operative interference from the interior of the uterus will be impossible; and dangerous, because inflammation is liable to follow, and that, too, in patients in the worst possible condition for resisting the attack.—*Med. Reporter.*

*Treatment of "Chronic Diarrhea" by Koumiss.*—In some cases the opium preparations, in others the mineral acids and vegetable astringents, or aqua calcis, etc., were sufficient to cure an attack or even the disease itself; but in other cases I battled in vain, although I have employed nearly all the good weapons of the Pharmacopœia; but it struck me particularly that these latter cases were especially those in which the appetite, digestion, and in some even the nutrition, were more or less impaired. In all of the cases I evidently lost ground with every return of the diarrhea. Being therefore compelled to look around for other preparations to combat these latter complications, I am happy to say I found in the old koumiss—of either sort of tull, medium, and whey-koumiss, according to the plumpness of the individual—the required remedy, which in a few weeks cured the chronic diarrhea, increasing at the same time the appetite and improving the nutrition. These latter properties of the koumiss are particularly advantageous in all complications with chest-diseases, in cases of excessive expectoration, in heart and kidney-diseases, and wherever anæmia, general weakness, and impaired digestion and nutrition prevail. Children with lymphatic constitutions, swollen abdominal glands, and relaxed mucous membranes, with scrofula and a general bad health, benefit very greatly by a koumiss treatment in a few weeks. In stout people I usually curtail the diet to very small but frequent meals of fish, eggs, meat in any form of cooking, and I allow them to drink as much old whey-koumiss as they may like; but the articles generally to be avoided during the treatment of chronic diarrhea, especially at the beginning, are milk, beer, sugar, vegetables and fruits; most of the condiments, as onions, garlic, mustard, pepper, vinegar; certain fats and oils, particularly oily fishes and birds. These restrictions are absolutely necessary, and are to be relaxed only when the digestion improves and the normal tone of the bowels returns, which shows itself in a normal frequency of the stools, and the normal shape, color, etc., of the fæces. In two or three months, and sometimes sooner, I find patients have nearly entirely been freed of these restrictions without fear of a recurrence of their chronic complaints.—*Jagielski in the British Medical Journal.*

*The Iodides in Lead-Poisoning.*—At a meeting of the Academy of Medicine of Paris, Dr. Faure communicated a note on the efficaciousness of the iodides in lead-poisoning. These observations had been made by the writer in a white-lead factory, his own property. He made the experiment on himself after a very prolonged period of poisoning, and a partial cure by the usual remedies; he obtained excellent results by a treatment of iodide of potassium, administered in doses of two centigrammes. After that time, and notwithstanding an excessive sensitiveness to saturnine emanations, he had always successfully overcome frequently repeated poisoning. M. Faure was ot opinion that a workman sufficiently intelligent to determine the quantities he ought to take, would always obtain the most satisfactory results by a treatmen consisting of doses from five to ten centigrammes of iodide of iron or potassium, without being obliged to interrupt his work.—*New Remedies.*

*Impotence.*—Dr. A. J. Jessup, New York, replies to Hallerus, to employ general hygienic treatment. Five-grain doses of bromide of iron formed into a pill with extract of gentian, four times a day. Then, should there be considerable irritability, give twenty grains of bromide of potassium, in solution, at bedtime, each night. Enjoin abstinence from conjugal contact for a number of weeks.

ANOTHER.—Dr. C. B., of Miss., replies: I would suggest the following treatment: I will preface it by saying that I have found the phosphide of zinc the most suitable and reliable form of administering that important element, phosphorus. I have recently (and successfully) used, in two such cases as described by your enquirer, the following:—

R. Zinci phosphidi,........ gr. ix
Extracti nucis vomicæ, gr. xxss.
Extracti conii,.............drachm iss
Ferri redacti,.............drachm iij
Vel. ferri phosphatis,...drachm vj.M.
Ft. pill No. 90.

Sig.—Take one pill three times a day.

At the same time, as adjuvants to this medication, apply galvanism to the lumbar region, and electricity to the prostatic portion of the urethra, by means of a urethral electrode, the negative pole being over the sacral plexus; also cold baths to the spine twice a day, and monobromide of camphor at night, in sufficient doses to control sexual excitement, taken half an hour before retiring.—*Medical and Surgical Reporter.*

*Chronic Eczema.*—J. S., aged 42, a baker and confectioner, very temperate man, always had good health, with the exception of an occasional attack of cracked fingers. He now suffered from severe eczema of all the phalanges of the left hand, which had been on him for several months. He was ordered to take three minims of liquor arsenicalis in half a wineglassful of water after each meal, to bathe the hands frequently in bran-water, and rub the fingers well with carbolized oil night and morning. He was completely cured in three weeks.

Mrs. W., aged 36, at present under treatment, is the mother of several children, of temperate habits, rather inclined to corpulency, but otherwise enjoys good health. She has had eczema of all the phalanges of both hands for more than two months. The fingers are very red and swollen, with numerous fissures, which are extremely painful, and discharge watery fluid. She was ordered to bathe the hands frequently with bran-water, and then cover them with lint constantly moistened with lotio plumbi. The inflammation quickly subsided, and the usual carbolized oil was substituted for a lotion. She is taking internally a saline aperient mixture, and is rapidly getting well.—*British Medical Journal*, Sept. 2, 1876.

*Hot Water in Croup.*—Dr. Dawasky. sanitary commissioner in Celle, refers to a remedy which has long proved efficacious in croup, but which seems of late to have fallen into unmerited oblivion. He has used the remedy since 1835, with the best results. The procedure seems to consist in allowing the hands and arms of the child to hang as deep as possible in a vessel of water. Hot water should be added frequently, and the application continued till the skin is swollen and reddened. After uncovering the arms and neck of the child as far as the breast, it is to be placed in the nurse's lap, in such a way that the arms hang deep into the hot water. A

cloth is then placed over the child's head and the hot-water vessel, so that it can inspire the warm vapor. When the arms have become intensely reddened and swollen the child breathes more freely and becomes sleepy. It is then dried carefully and put to bed. Profuse perspiration and disappearance of the unpleasant symptoms promptly follow if the remedy has been applied at an early stage of the disease. It is no longer of use when the membranous formations have occurred. —*Memorabilien.—N. Y. Med. Jour.*

*On the Treatment of Ranula.* —The Paris correspondent of the *Brit. Med. Jour.*, in his last letter, draws attention to the treatment of ranula, and alludes to the success attending injection of chloride of zinc, as practiced by M. Panas. I might observe, without entering into the morbid conditions leading to obstruction of a sub-lingual gland or duct, that, practically, the surgeon's intention is to make a permanent opening in the sac, one which will allow the saliva continuous and natural exit into the mouth. It occurred to me, some years ago, that the use of a metalic seton, acting, to some extent, as a drainage-tube, would attain this object; and as two cases (both children) came under my notice, I tried the following operation. An ordinary suture-needle, carrying medium-sized silver wire, having been passed directly through the sac-like tumor from one side to the other, the ends of the wire were brought forward, twisted together, and cut off, leaving a small ring of metal half within and half externally. The wire was allowed to remain three weeks, then cut and withdrawn. It caused no irritation or impediment, and a patent orifice remained after removal. Both cases were permanently cured.

*Protagon.*—Dr. Polk, in an article on this new agent (*New Remedies*), says: "Miss A. M. P., of Delaware, has been a patient of mine for four years. This lady, forty-three years of age, had been in ill-health nearly twelve years; has had cough, diarrhœa, night-sweats, and four months ago was emaciated to a mere skeleton. Heretofore she refused to take the hypophosphite of oleine, on the plea that it sickened her stomach and increased the diarrhœa; but finding herself nearly approaching the grave, she agreed to try it once more. I prescribed the following:

Protagon (from the brain of
the cow)........................ dr.vj.
Alcohol..............................oz.j.
Oil of Eucalyptus ...... ........oz.ij.
Cod-liver oil (Norwegian)......oz.iij.
M. Take a desertspoonful twice daily.

The purpose of the oil of eucalyptus was to cover the taste of the cod-liver oil, which it does effectually, but the cost is a barrier to its general employment; otherwise cod-liver oil could be rendered a very agreeable medicine. Under the above treatment the diarrhœa and night-sweats disappered, her appetite returned, digestion became excellent, and to-day she is visiting my house, declaring herself entirely well. She is fleshy and the very picture of health,

*Tic Doloreux of the Face Cured by Bromide of Potassinm.*—Dr. Petes narrates a typical case of this formidable malady cured by the bromide of potassium. The pain occupied the supra-orbital branch, the superior and inferior maxillary divisions of the fifth nerve, and had the characteristic severity of that form of *tic* entitled by Trosseau *epileptiform neu-*

*ralgia.* Entertaining the view that these cases are really a mode of expression of the *status epilepticus*, Dr. Peter came to the conclusion to administer bromide of potassium in large doses—6 *grammes*, about 95 grains a day—for several weeks. This treatment was crowned with success. She slept well the first night, and three or four days after the use of the bromide had begun, the pains had completely disappeared. The remedy was continued for three months, as follows:

| | | | |
|---|---|---|---|
| 1st month | ......6 grammes, | about | 95 gr. |
| 2d " | ......4 " | " | 64 " |
| 3d " | ......2 " | " | 32 " |

—*Bull. Gen. de Therap.* 30th October, 1867.—*Clinic.*

[We have found the bromide, combined with tincture gelseminum, to be a good remedy in these cases. Ed. Rec.]

*To Check Colliquative Sweating.*—The exhaustive sweats in surgical diseases and phthisis are entirely controlled, according to Dr. Thomas J. Dunott, of Harrisburg, Penn., by small hypodermic injections of atropia, and sponging with hot vinegar. In a case in point, given in the *Virginia Medical Monthly*, he writes of a case of osseous injury: "He sweats profusely and constantly; to have ice pills and hypodermic injections of one-hundredth of a grain of atropiæ sulph·; also to be sponged with hot vinegar. This controlled the sweating, which was so profuse as to keep the bed-clothing saturated whenever the atropia and sponging were omitted. It is my belief that a very small dose of atropia, when combined with the hot vinegar application, will be most effective in controlling this exhausting discharge from the skin. Neither used alone would be successful; but my experience with atropia is limited to doses no larger than the one mentioned—one-hundredth of a grain."—*Med. and Surg. Rep.*

*Raw Onion as a Diuretic.*—Dr. G. W. Balfour, in the *Edinburgh Med. Jour.*, records three cases in which much benefit was afforded patients by the eating of raw onions in large quantities. They acted as a diuretic in each instance. Case first was a woman who had suffered from a large white kidney and constriction of the mitral valve. Her abdomen and legs had been tapped several times, but after using onions as above she had been free from dropsy for two years, although still suffeirng from albuminaria. Case second suffered from cardiac disease, cirrhotic liver, and acites. Case third had ascites depending on tumor of the liver. In both of them the remedy had been used with good results. Both had been previously tapped, purgatives and diuretics alike having failed to give relief. All other treatment having failed to give aelief, recourse was had to the onions. Under their use the amount passed steadily rose from ten to fifteen ounces to seventy-eight or a hundred.

*The Relief of Prickly Heat.*—Persons subject to this annoying affection will be glad to learn that Surgeon Major Dr. J. G. French, of the Indian medical ser vice, in a contribution to the *Indian Medieal Gazette*, says that we can cure prickly heat in three or four days by the application of a solution of sulphate of copper· This should be of the strength of about ten grains to the ounce of water, and the solution should be applied daily, or oftener, by means of a camel-hair brush or bit of sponge tied on the end of a stick It is best applied after the morning bath, when the skin has been well rubbed with the towel, and it must be allowed to dry on the skin before dressing. Dr. French states that he has used this application for over thirteen years, and when regularly and properly applied, he has never known it to fail.

*New Anæsthetic Agent.*—Rabuteau, in a memoir read before the *Acadcmie des Sciences*, states that he has investigated the physiological properties and mode of elimination of hydrobromic ether. He has satisfied himself that this anæsthetic agent, which possesses properties intermediate to those of chloroform, bromoform, and ether, might be advantageously employed to produce surgical anæsthesia. The hydrybromic ether is neither a caustic nor an irritant. It can be ingested without difficulty; and applied without danger, not only to the skin, but to the external auditory meatus and to the mucous membrane. It is eliminated completely, or almost completely, by the respiratory passages, in whatever way it may have been introduced into the system.—*Med. and Surg. Rep.*

*Antihydropin.*—Dr. Bogamolow some time ago discovered in cockroaches (*Blatta orientalis*, Orthoptera) a crystalline substance, which he named antihydropin, from the favorable effects obtained by him with it in the treatment of dropsy. Roaches are highly esteemed as a popular diuretic by the common people in Russia ; this fact induced Dr. B. to employ them in various forms, such as decoction, tincture, and powder, and in the form of the supposed alkaloid. Under its use the amount of urine increases, albumen and casts diminish in quantity; œdema of hands, feet, and face subsides, the weight of the body increases, and the pores of the skin begin to act more freely. The remedy is said not to interfere with digestion, nor to irritate the kidneys.—*Petersb. Med. Woch.* in *Ph. Z. f, Russl.*

*A Combination of Tœniafages.*—Take of pomegranite bark, oz.ss ; pumpkin seeds, oz.i; ethereal oil of male fern, dr.i; ergot (freshly bruised), dr.ss; powdered gum acacia, dr.ij ; croton oil, mij. Upon the pomegranate, pumpkin seeds and ergot, pour eight ounces of water. Bring to the boil, stirring constantly whilst boiling for fifteen minutes; adding water to keep up to eight ounces. Make a smooth emulsion with a small quantity of water, of the croton oil, oil of fern, and gum acacia. Strain the decoction through a coarse cloth, and express strongly, and mix with the emulsion.

The patient should have a full dose of aperient (Rochelle salts, oz.i) on going to bed; and the following morning the above dose about eight o'clock, before any food·—*Brit. Med. Jour.*

*Hay Fever*—Dr. Brinton in Philadelphia Medical Society (*Med. Rep.*) says of hay fever, that " As it may come on at any time during the summer, the term hay fever is a misnomer ; in susceptible individuals dust will cause the disorder at any season of the year. It is a neurosis connected with constitutional idiosyncrasy, dependent upon a cause whose effects are invariable and not susceptible of cure, the only means of relief being the removal of patients from the neighborhood of the irritant. He has never seen a case cured, and Dr. George N. Beard, the author of an excellent monograph on this subject, has expressed the same opinion."

*Ergot in Typhoid Fever.*—Dr. Duboue, of Pau, France, has recently related his experience in the treatment of typhoid fever by ergot. What next will this universal specific be credited with curing? He gave it to eight men and seven women. There were two deaths—the others recovered, though some were admitted into the hospital in the last stage of the affection. Now we should like to know if the ergot contributed any impulse towards a cure? If it did, then on what physiological principle? Typhoid fever is classed as a zymotic disease· the blood is contaminated with a special virus, and ulceration of the bowels is its constant and almost pathognomonic symptom. How would ergot meet such a case?

*An Emmenagogue Pill,* the formula for which is given in the *Union Medicale,* may be made as follows:

R. Aloes ................gr.xv.
Rue,
Saffron,
Savin ............aa gr.vijss.

M. Divide into 10 pills, one of which is to be taken morning and evening, commencing two or three days before the supposed menstrual epoch. It is desirable to employ, in connection with the pills, warm hip-baths, dry cups to lumbar regions, leeches to the upper and inner portion of the thighs, and walking exercise; also give in the menstrual intervals, iron and quinia.

*Jaborandi as a Galactagogue.*—This new medicine has been proven to be a powerful sialagogue and diaphoretic, and now a writer in the British *Medical Gazette* announces that he has found it to possess great energy in promoting secretion from the mammary glands, and bringing on a copious supply of milk after other approved means have failed. The dose used was five grains of the powder three times a day, infused in warm water. The medicine is considered, even if it can do no good, to do no harm to either the infant or its mother.

*Subcutaneous Injeetions of Cold Water in Acute Rheumatism.*—Dr. Dieulafoy has, for several years past, been in the habit in acute articular rheumatism, of injecting some ten drops of cold water around different parts of the affected joint as a means of relieving the pain. The results are most remarkable. The pains abate and the patient is enabled to move the joint, and in some cases the rheumatism is even cured by this simple means. The same means may be employed also in muscular rheumatism, ischias, etc.—*Med. Times and Gazeette,* January 27, 1877.

*Carbolic Spray in Bronchial Catarrh.*—Dr. Moritz communicated to the St. Petersburg Medical Society the results of his trials of carbolic acid spray in various forms of bronchial catarrh, relating several examples of its utility. Since he had much to do with this spray he found that bronchial catarrh, to which he was formerly much subject, either ceased to appear or was soon cut short. In as small a room as possible he causes half a pound of a 2 per cent. solution of the acid to be sprayed per diem, the night being the time especially to be preferred.—*St. Petersburg Woch.*, November 11.

*Junctus Acutus in Ascites.*—The list of diuretics employed in this complication is not small; but it would appear that a French surgeon, residing in Algeria, has been very successful with *Junctus acutus L.* About twenty stems should be subjected to decoction, both the white and green parts being used. It may be mentioned that M. Cazin has found similar properties in an indigenous plant (the *Butomus umbellatus L.*). One ounce of the root is to be boiled in a quart of water.—*New Remedies.*

*Laxative Antibilious Pills.*—

R. Extr. Colocynth Co.....grs. lxxvii.
Scammony.....................grs. xix.
Extr. rhei..............................grs. xi.
Saponis alb... .. . .........grs. ivss.
Essent. canell..................gtta. iv.

M.—ft. mass and divid. in pilul, No. 24.

Sig.—One or two pills to be taken morning and evening—to provoke bilious stools and augment the appetite—in gastric embarrassment.—*Progr. Med.*

*Potion* against the symptom of oppression in the nervous or inflammatory affections of the respiratory tract:

R. Infusion of Polygala...oz. iii.
Nitrate of potass..dr. i. gr. xv.
Syr. belladonna..........oz. i.
Tinct. lobelia........gtt. xxx.
M.

Sig. A tablespoonful every hour, and when the oppression ceases a tablespoonful every two hours. This potion may be employed in affections of the right side of the heart, for the purpose of lessening the oppression.—*Progress Medicale.*

*Fetid Feet.*—M. Ortega advises to apply compresses soaked with a solution of chloral, which has the effect of destroying the smell and curing the ulcerations of the sole.

*Chromic Acid for Warts.*—Three or four applications of this acid will cause the disappearance of warts, however hard, large or dense these may be.—*L'Union Medicale.*

*Treatment of Acute Articular Rheumatism by Cyanide of Zinc.*—This remedy, suggested some time since by Luten, of Rheims, has lately been employed with success by Deschamps, who recommends the following formula:

R. Zinci Cyanidi ................gr. 1/12

Pulv. acaciæ,

Sacch. lactis..............aa q. s.

M. Ft. in pil. j.

To be taken to the number of ten in twenty-four hours.—*New Remedies.*

*Formula for Diphtheria.*—

R. Sulphocarb. sodii........dr. ij.
Quiniæ sulph...............dr. j.
Acid sulph. aromat......dr. j.
Syr. aurantii cort...ad. oz. iv.
M.

A teaspoonful to be given every three hours. Child five years old.

The throat to be showered, by means of a syringe, cnce an hour with a mixture of chlor. potass. mur. tinct. ferri and water.

*The Cold Douche in the Treatment of Buboes.*—In a recent discussion in the Medical Society of Dresden, Dr. Kuntzelmann stated that it was his custom to employ the cold douche early in the treatment of ordinary buboes, and he had in numerous instances succeeded in preventing suppuration.—*Schmidt's Iahrbucher*, No. 10, 1866.

*Colored Spectacles*—Dr. Magnus condemns the use of blue glasses as a protection for the eyes; and prefers the gray and smokey glasses used in England. He considers blue glasses specially irritating to the eye, and says that many birds, reptiles, and amphibians have yellow or reddish oil drops in the eye to neutralize this blue color and protect the eyes.

*Antidotes of Arsenic.*—(*a.*) Hydrated sesqus oxide of iron recently prepared (gelatinous and brown) is an antidote for arsenious acid, but not for the arsenate of potash, nor for the arsenate of soda. (*b.*) At a longer interval than an hour it is useless to attempt recovery from poisoning by arsenic. (*c.*) For arsenite of potash, and arsenite of soda the author proposes perchloride of iron in conjunction with magnesia. (*d.*) The mode of administration is the official solution of perchloride of iron, and a half an hour after magnesia in the proportion of a drachm to 2⅝ ozs. of perchloride. (*e.*) This perchloride of iron and magnesia are also an antidote for arsenious acid. Therefore, it is preferable to employ it always in cases of poisoning by arsenic or its compounds. (*f.*) An hour after the administration of an atidote, it will always be well to employ a purgative, in order to expel the ferrated arsenite which is formed, and as this arsenite is soluble in acids, to avoid acid drinks and lemonades.—*Canadian Jour.*, Sept. '76.

*Salicylate of Soda in Rheumatism.*—The formula used is as follows:

R. Acid salicylic..............dr.iij.
Sodæ bicarbonate..........dr.ij.
Glycerine,
Aq.........................aa oz.ij.

M. Sig. Tablespoonful every two hours for the first day, and afterward the same dose six times a day.

*Nitric Acid for Hoarseness.*—Dr. W. Handsell Griffiths says that a few drops of nitric acid in a glass of sweetened water, a couple of times daily, will be found an excellent remedy for the hoarseness of singers. One of the largest fees ever received by him—so he says—was for this prescription.

*Hot Packing in Rheumatism.* — We have recently tested the method of hot packing in a case of acute articular rheumatism, with very great relief to the patient's sufferings. Blankets rung out of hot water were applied to the affected parts, with bottles of hot water to keep them from cooling off, and thus avoid the too frequent removal of the cloths. This, with the following internal remedy was mainly relied upon, the case recovering in about ten days:

R. Muriate of ammonia......oz.ss.
Water.........................oz.xii.

Dose, one tablespoonful, alternating at four hours with salcin, grains x. Every other night a dose of com. liquorice powder was given as a purgative.

W.

*Safrol.*—Crystalling oil of sassafras was first examined by St. Evre in 1844, under the name of sassafras-camphor. According to him the crystals melt between 5° and 17° C., and congeal again at 7-5° C. Grimaux and Rouette, who examined the oil in 1869, do not mention this crystalline portion, although found a substance having the same boiling point and elementary composition as sassafras-camphor, but not becoming solid even at 20°. They named this substance safrol.

*Chloral Plaster.*—Dr. Solari, of Marseilles, says the *Medical Examiner* recommends the chloral plaster as an excellent application in cases of neuralgia, and of pains resulting from exposure to cold. The plaster is easily prepared by powdering the chloral over a common pitch plaster, one or two scruples of the chloral for every four square inches of plaster, care being taken not to incorporate the chloral with the pitch.

### TINEA TONSURANS.

This disease, *porigo furfurans* or *ringworm of the scalp*, is thus explicitly defined in Prof. Duhring's work on Diseases of the skin, a review of which may be seen in our present issue: " *Tinea tonsurans is a contagious, vegetable parasitic affectiou of the scalp, due to the Trycophpton. characterized by one or more circular, variously sized, scaly, more or less bald patches, showing the hair to be diseased and broken off close to the scalp, accompanied by itching.*"

The treatment suggested is, in substance, as follows: The part being washed daily with soap and water and dried, the loose hairs about the edges of the patches, and the stumpy, broken off hairs over the surface, should be extracted with forceps, a few at a time each day and a parasiticide applied. A solution of corrosive sublimate, 2 grains to the ounce will usually be found effecttive. Or, in lieu of this the following preparation (much used in the London hospitals) may be used:

R. Iodine .......................... dr.ij.
Oil of tar..................... oz.i.

Mix slowly and well, and apply once every three or four days.

*Small Poisonous doses of Chloral in Delirium Tremens.*—Dr. Frank, of the Cologne Burger-Hospital, after exhibiting, by means of a long array of references, the great discrepancy existing among writers on the employment of anæsthetics with respect to the maximum and poisonous doses of chloral, observes that these really much depend upon the peculiarities of the patient, who even may himself differ at different times upon this point. In delirium tremens very small doses sometimes produce fatal effects and he relates two cases which have occurred to himself. Both were men in the prime of life, with no disease of any organ, as shown at the autopsies, and yet in both small doses (one gramme and a quarter in one case, and two grammes in the other) of chloral of undoubted purity produced fatal paralysis of the heart. — *Berlin Klin. Woch.*, September 11.

*Therapeutic Effects of Corrosive Sublimate in Blumorrhœa.*—Dr. Leopold Bruck, of Buda-pest, states that he has found blumorrhœa urethræ, lasting as usual when injections are employed six weeks without complication, to be curable by the administration of corrosive sublimate. The discharge is profuse during the first two days, but subsequently becomes progressively less abundant and more serous; the sensation of burning in the urethra is bearable, and the chordee moderate. During the treatment, alcoholic fluids, coffee, and highly seasoned food must be avoided. Purgatives should be excluded, since they are unnecessary during the use of the sublimate. The remedy is apt to produce pain in the stomach and intestines, and if this occur its use should be omitted for a few days. It should not be given in cases of cardiac and pulmonary diseases. Dr. Bruck prescribes the sublimate in the form of pills.—(*Centralblatt f. d. med. Wiss.*—*The Practitioner*, December, 1876.)

### GLEET AND CHRONIC GONORRHŒA.

As an injection in gleet and chronic gonorrhœa use the following:

R. Hdrastin canadensis.......dr.ij.
Deod. tinct. opi.. .........dr.j.
Aquæ,
Mucilag. acaciæ.........aa oz.ij. M.

*Arsenic in the Stomach.* — 1st. Grains 0.926 of arsenious acid in solution to the kilogramme (2⅝ lb.), the weight of dogs, injected into the stomach, is enough to cause death in nearly all cases. 2d. The dose of grains 1.08 to the kilogramme is certain to cause death. 3d. If poisoning supervened only on administration of a stronger dose, it was much more rapid, and this being relative to a particular condition in dogs, which throws off the poison too quickly. 4th. In poisoning by the average dose of grains 0.926, death ordinarily takes place at the end of 24 hours.

---

*Bromide of Potassium in Hæmorrhage.*—It has been asserted that this agent induces contraction of the blood vessels, and Dr. Geneuil (*L' Union Medicale*) has used a local application in the treatment of epistaxis, uterine hæmorrhage, and coryza. It should also be given internally. In coryza two injections of a saturated solution into the nose give relief in an hour, and a permanent cure is effected in about six hours.

